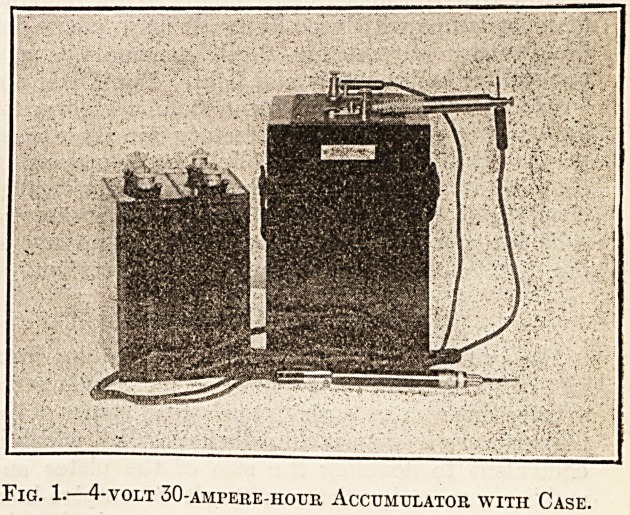# Electricity in Modern Medicine

**Published:** 1911-12-09

**Authors:** Alfred C. Norman

**Affiliations:** House Surgeon at Sunderland and Durham County Eye Infirmary; late House Surgeon in charge of *x*-ray Department, Royal Buckinghamshire Hospital.


### ELECTRICITY IN MODERN MEDICINE.

### II.—Sources of Electricity.

(*Continued from p.* 202.)

By ALFRED C. NORMAN, M.D. Edin., House Surgeon at Sunderland and Durham County Eye Infirmary; late House Surgeon in charge of *x*-ray Department, Royal Buckinghamshire Hospital.

ACCUMULATORS.—Accumulators, or storage batteries, are so convenient for medical men who have no electric-lighting supply, and they are so efficient when portability is essential and fairly large currents are required that they merit consideration in some detail. They have to be charged at least once in six weeks, even when not in use, but nowadays it is generally possible to find within reasonable distance a motor garage or power station where recharging is done.

The discharge of electricity from an accumulator takes place as a result of chemical action, but the elements are so chosen and arranged that when the cell is nearly exhausted the chemicals can be restored to their original form by passing a current of electricity through them in a reverse direction from their own discharge.

### How to Work an Accumulator Cell.

In describing an accumulator cell the plates attached to the positive (+) and negative (−) terminals will be termed the + and − plates respectively. It is well to remember that the fully charged + plate is of a dark chocolate colour and that the + terminal is usually painted red by the makers, while the − plate has a greyish or "leaden" appearance and its terminal is distinguished by being painted black.

A type of cell which is frequently made contains two plates attached to the + terminal and three attached to the — terminal, so arranged that a + plate is suspended between two — ones to utilise both sides of the + plates. The electrolyte is a solution of pure sulphuric acid (about 1 part to 4 by measure) having a s.g. of 1.200. When such a cell, fully charged, comes from the makers, the + plate consists of a grid, made of lead, the interstices of which are filled with a paste of red peroxide of lead ($PbO_2$), while the — plate is a similar grid whose interstices are packed with metallic lead in a state of fine division, i.e. "spongy lead." When we connect the terminals of the cell with a conducting wire, forming an external circuit, chemical re-action is set up between the acid and the plates; the peroxide at the + plate is gradually reduced to lead sulphate, and transfers a charge of electricity to the fluid, while the "spongy" lead at the — plate is *oxidised*, also to lead sulphate, and likewise transfers a charge of electricity to the exciting fluid, the result being that we get a flow of current in the external circuit from the + to the — terminal.

When the cell requires to be re-charged we may pass a current from the main through it from the + to the — terminal. This charging current liberates oxygen at the + plate, which oxidises the sulphate there into peroxide of lead, while hydrogen is liberated at the — plate, and reduces its sulphate to "spongy" lead. When the process is complete oxygen and hydrogen escape in the form of tiny bubbles. The process, then, of charging an accumulator simply consists in changing the chemicals back to their original condition, in which they are again capable of giving rise to electrical energy.

Accumulators should be tested frequently with a voltmeter (a pocket size of which can be obtained for about 8s.), and they should be re-charged immediately the voltage drops below 2 for each cell. If they are left in a low state for any length of time they become "sulphated," *i.e.* the sulphate of lead on the plates becomes insoluble, and they never regain their original efficiency. Even when they are not in use accumulators tend to "sulphate" if left uncharged for longer periods than six weeks. The writer always re-charges his on the first day of every month, whether they have been used or not.

### The Advantages of Accumulators.

Compared with primary batteries accumulators have many advantages. If we connect the terminals of a 1.5-volt primary cell with a wire of known resistance the current flowing through the wire would be less than Ohm's law would lead us to expect, because there is a certain resistance in the contents of the cell which we term "internal

FIG. 1.—4-VOLT 30-AMPERE-HOUR ACCUMULATOR WITH CASE.

resistance." For primary batteries Ohm's law would read: $C = \frac{E}{R+r}$, where R is the resistance in the external circuit and $r$ the internal resistance of the cell. The larger a cell is made the smaller its internal resistance becomes. Further, on account of polarisation we find that the current from a primary cell tends to diminish after it has been flowing for a short while, even though the external resistance remain constant. Now accumulators have none of these disadvantages: their internal resistance is so small (from .001 to .006 ohm) that we can ignore it for most purposes, there is practically no polarisation and the voltage of each cell remains almost constantly at 2; consequently they give a larger current for their size than any primary battery and their discharge does not fall off until they are almost exhausted. An accumulator must not at any time be allowed to discharge more ampères than the size of its plates will stand or they will be disintegrated. A safe current for a cell of 30 ampère-hour capacity might ruin the plates of a 5 ampère-hour cell, and no cell would stand being short-circuited, for it would then be able to discharge all its current at the rate of hundreds of ampères per second.

### Improvements Due to Motor-cars.

Thanks to the advent of the motor-cycle and car, accumulators can now be obtained in celluloid cases with sealed tops, an arrangement which renders them much lighter and reduces to a minimum the possibility of spilling their acid contents. These ignition accumulators are admirably adapted to medical requirements and they are comparatively cheap. Their capacity is nominally marked in ampère-hours, but it must be remembered that this is only for ignition purposes (*i.e.* interrupted discharges of about ½ ampère), and that the total number of ampères an accumulator will give out decreases very markedly if we make the discharge heavier. For instance a 4-volt 30-ampère-hour accumulator of this kind will give a continuous current of ½ ampère for about 40 hours before it has to be re-charged, whereas it would only give 1 ampère for 16 hours or 2 ampères for 6 hours.

Now that the makers can supply 4-volt metallic filament lamps with practically all instruments for illuminating purposes, a 2-cell accumulator can be used for both cautery and light. The 4-volt 30-ampère-hour variety made by an Edinburgh firm for motor ignition is very satisfactory for these purposes. It is supplied in a celluloid case measuring about 3¾ in. by 3 in. by 5½ in. high, and weighing about 6½ lb. The price is 23s. When the two cells are connected in series it will light up any of the 4-volt lamps used for medical examination.

### Currents for Cauteries.

For ordinary cautery purposes we require from 12 to 18 ampères, but 2 volts will more than serve to drive this current through the low resistance of the burners, so we connect the cells in parallel, which is equivalent to doubling the size of the plates and making our accumulator a 2-volt 60-ampère-hour one. The advantage of this arrangement is that the plates will now stand the heavy discharge and that the accumulator will give out twice the total amperage it would have done in series.

It is an advantage to order with the accumulator a wooden carrying case with leather handle, and to have, fixed to the lid of the case, a switch devised to put the cells in series or parallel at will. Fig. 1 shows a 4-volt 30-ampère-hour accumulator with carrying-case and the writer's own pattern of switch for this purpose.

### Recharging.

At most garages they will charge a 30-ampère-hour accumulator for 6d. or 9d., which is much cheaper than it can be done at home, because at a garage they usually charge several in series at a time.

If we wish to charge our accumulator from the continuous-current house mains we must remember that its internal resistance is so low that if connected directly to the mains there would be a sudden rush of current, which would spoil the plates and blow the fuses of the installation. We therefore use one or more carbon lamps as a resistance to regulate the flow of current. A current of about two ampères will charge a 30 ampère-hour accumulator in from eight to fifteen hours, depending upon how much use it has had. This current would be allowed to pass by a 60-candle-power carbon lamp on a 110-volt supply, or by two 60 c.p. lamps in parallel on a 220-volt supply.* If our supply happens to be a continuous one of 220 volts, we ask our electrician to make up a small charging board having two 60-c.p. carbon lamps in parallel, a plug adapter, a safety fuse, and two free wires to connect to the accumulator. *It is essential to connect the + wire of the supply to the + terminal of the accumulator* and the — wire to the — terminal. To make sure of doing this, we insert the plug adapter into a convenient lampholder and turn on the switch; we then take the two wires (holding them by their insulated covering to avoid a shock), and apply them, about a quarter of an inch apart, to a piece of wet, blue litmus paper; the + wire at once turns the litmus *red.* We then connect the wires to the terminals of the accumulator and allow the current to flow until gas is given off freely at both plates. When charging is complete, the acid is so full of small bubbles of gas that it is usually described as being "milky;" it is a waste of current to continue the process.

There is a celluloid plug in the top of each cell, which should be removed before starting to re-charge. The acid must always cover the tops of the plates. To make up for loss by evaporation a little distilled water must be added from time to time, and very occasionally a little more acid, having a s.g. of 1.200.

* It must be noted that the above examples apply to the *continuous* current *only*, and that the lamps used are 110 and 220-volt ones respectively. To charge accumulators from an *alternating* current supply we must use a current rectifier—a troublesome and expensive piece of apparatus.

(*To be continued.*)

## Figures and Tables

**Fig. 1. f1:**